# Statement of the ASPHER Task Force on War and Public Health on the Conflict in Israel/Palestine

**DOI:** 10.3389/phrs.2024.1607047

**Published:** 2024-02-23

**Authors:** Oliver Razum, Paul Barach, Tomasz Bochenek, Colette Cunningham, Nadav Davidovitch, Polychronis Kostoulas, Jutta Lindert, Henrique Lopes, Vladimir Prikazsky, John Reid, Mirjana Kujundžić Tiljak, John Middleton

**Affiliations:** ^1^ Department of Epidemiology and International Public Health, School of Public Health, Bielefeld University, Bielefeld, Germany; ^2^ ASPHER Task Force on War and Public Health, Brussels, Belgium; ^3^ College of Population Health, Thomas Jefferson University, Philadelphia, PA, United States; ^4^ Institute of Inflammation and Ageing, Queen Elizabeth Hospital, University of Birmingham, Birmingham, United Kingdom; ^5^ Institute of Public Health, Faculty of Health Sciences, Jagiellonian University Medical College, Krakow, Poland; ^6^ School of Public Health, University College Cork, Cork, Ireland; ^7^ School of Public Health, Ben-Gurion University of the Negev, Be’er Sheva, Israel; ^8^ ASPHER Public Health Emergencies Task Force, Brussels, Belgium; ^9^ Faculty of Public and One Health, University of Thessaly, Volos, Greece; ^10^ Department of Health and Social Work, University of Applied Sciences Emden/Leer, Emden, Germany; ^11^ NOVA Centre of Global Health, NOVA-IMS, NOVA University of Lisbon, Lisbon, Portugal; ^12^ School of Allied and Public Health, University of Chester, Chester, United Kingdom; ^13^ Andrija Stampar School of Public Health, University of Zagreb, Zagreb, Croatia; ^14^ University of Wolverhampton, Wolverhampton, United Kingdom

**Keywords:** conflict region, war, public health, ASPHER, peace

The Association of Schools of Public Health in the European Region (ASPHER) reaffirms its condemnation of the terrorist attacks by Hamas that killed or abducted several hundred civilians in Israel on 7th October 2023 [1]. We call attention to the statement by the chief prosecutor of the International Criminal Court (ICC) who said that the atrocities committed by Hamas were “some of the most serious international crimes that shock the conscience of humanity,” and that the ICC is ready to prosecute those responsible [2]. We also call attention to the Hamas Covenant of the Islamic Resistance Movement, originally issued on 18 August 1988, that calls for the destruction of Israel, killing all Israelis, and calls for jihad on the Jewish people [3]. As public health professionals we condemn dehumanization and acts of genocide. We support Israel’s right to armed self-defence within the limitations of international humanitarian law [4]. At the same time, we emphasise that Palestinians have legitimate aspirations to live with equal measures of security freedom, justice, opportunity, and dignity [5]. This applies to all the people of the region and beyond.

We urge the immediate and unconditional release of all hostages held by Hamas. We particularly condemn all gender-based violence [6] and support UN calls for an investigation of the numerous accounts of sexual violence perpetrated by Hamas [7]. At the same time, we express our grave concerns about the millions of displaced people in Gaza and Israel as a consequence of the war. We call for all displaced persons to be enabled to return to their homes, with adequate support. We are concerned about the destruction of Gaza, in particular of hospitals and other civilian infrastructure, and the loss of life which has been inflicted on the people of Gaza, including women, children, and other non-combatants [8]. We are alarmed by the lack of water supply and sanitation, increasing the risk of communicable disease outbreaks. While we recognize the State of Israel and Palestinian jurisdictions, we assert that international bodies will need to sit in judgement on the conduct of Hamas and Israel as it relates to following international humanitarian law and the Geneva Convention [4].

We believe the current humanitarian catastrophe is a testimony to years of neglect and abandonment of the people who should have been served by their governments. There has also been a failure on the part of the regional and global community. All could have done more to actively pursue peace and security for Israelis and Palestinians, fulfil their legitimate rights and aspirations, and enable harmonious development.

We believe there must be rapid, visible diplomatic efforts to create trust-building practical steps towards peace and security for both sides. The conditions of ceasefire must be built upon, and massive humanitarian efforts must be allowed into Gaza, to enable urgent treatment for wounded civilians, and towards rehabilitation and recovery for the civilian population. There must be guarantees for the security of Israel who continues to suffer daily rockets and missile attacks. There must be moves towards a UN-brokered multi-national peace keeping effort in the region.

ASPHER is a public health organisation with more than 120 member schools including in Israel and Palestine. Our primary responsibility in war as in peace is saving lives. Public health leaders have moral and professional responsibilities to speak out about conflicts, and to contribute to prevention, limitation, and resolution of conflicts [9, 10]. Peace is more than the absence of war. Peace is an active process which requires all parties in conflict to desist from violence, and to seek peaceful solutions to their disagreements.

ASPHER is deeply committed through its members to assist all people and organisations affected by the conflict who are seeking to save lives and restore conditions for security and development. ASPHER greatly values colleagues in schools of public health in the region. We know that you have in the past sought to work harmoniously together in the interests of health and peace. ASPHER will make resources available to build bridges between Israelis and Palestinians. As health professionals we advocate diversity and respect [11]. We vigorously oppose antisemitism, anti-Muslim hatred, gender-based and sexual violence, and all other hate-based violence. As Schools of Public Health, we commit to developing the curriculum and competencies for the role of public health in the prevention of violence, for response, rehabilitation, and recovery after conflict. We commit also to supporting our students and our staff by speaking up in the face of injustice and in protecting them from the harmful impacts of conflict and enabling them to play their roles in the public health response to violence.
